# Evaluation of the Frequency and Anatomy of Radix Entomolaris and Paramolaris in Lower Molars by Cone Beam Computed Tomography (CBCT) in Northern Iran, 2020-2021: A Retrospective Study

**DOI:** 10.7759/cureus.46854

**Published:** 2023-10-11

**Authors:** Hadiseh A Rokni, Mona Alimohammadi, Narjes Hoshyari, Jamshid Y Charati, Amirmohammad Ghaffari

**Affiliations:** 1 Department of Endodontics, Mazandaran University of Medical Sciences, Sari, IRN; 2 Department of Oral & Maxillofacial Radiology, Mazandaran University of Medical Sciences, Sari, IRN; 3 Department of Endodontics, Faculty of Dentistry, Mazandaran University of Medical Sciences, Sari, IRN; 4 Department of Biostatistics, Faculty of Health, Mazandaran University of Medical Sciences, Sari, IRN; 5 Student Research Committee, Faculty of Dentistry, Mazandaran University of Medical Sciences, Sari, IRN

**Keywords:** radix entomolaris, radix, entomolaris, paramolaris, endodontic treatment, root canal therapy

## Abstract

Introduction and objective: The presence of an additional root, known as a radix, in the lower molars is of significant importance in the context of root canal therapy since it has the potential to contribute to treatment failure. Furthermore, it is imperative to take this circumstance into consideration when doing tooth extraction using a radix. The present study investigated the anatomical characteristics and prevalence rates of radix entomolaris and paramolaris in mandibular molars using cone beam computed tomography.

Materials and methods: Cone beam computed tomography scans of the lower molars of 376 patients were processed through Newtom’s NNT viewer software. Radix type, radix root canal length, radix root curvature, Vertucci's classification of the canal, and gender of patients were recorded. The results of the research were analyzed with chi-squared.

Results: The prevalence of radix was found to be 0.74%, with entomolaris and paramolaris present in 54.54% and 45.46% of cases, respectively. There was no statistically significant difference between the two variables of radix type and gender, as indicated by a p-value of 0.08. The mean curvature and length of the radix root were measured to be 56.63° and 15.09 mm, respectively. The average root curvature in individuals diagnosed with radix entomolaris and paramolaris was found to be 62.33° and 49.80°, respectively. The study found that the root curvature of entomolaris exhibited a statistically significant difference compared to paramolaris (P=0.031). The mean length of the radix entomolaris and paramolaris roots was found to be 15.50 and 14.60 mm, respectively. There was no statistically significant difference observed in the mean root lengths of the various radix types (P=0.37). According to Vertucci's classification, all radixes were classified as type 1.

Conclusion: The investigated population had a low incidence of radix. The curvature of radix entomolaris exhibited a statistically significant increase compared to radix paramolaris. There was no observed correlation between gender and the length of radix roots in relation to the type of radix root.

## Introduction

The mandibular first and second molars in the permanent dentition often display a dual-root morphology comprising a mesial and distal root. These teeth are frequently found to have three or four root canals. Occasionally, an additional tertiary root, the radix, may arise due to variances in ethnic heritage or dental morphology. The root stated above is primarily located in the distolingual entomolaris and paramolaris, with an occasional presence in the mesiobuccal [1,2]. The occurrence of a third root, primarily observed in the first molar rather than the second molar [3], can appear either as a separate entity or as an extension of other roots. The tertiary root is characterized by its smaller size relative to the primary roots and significant curvature and requires careful attention during root canal therapy [2]. There is variability in the occurrence of Radix entomolaris (RE) among various populations. It is worth mentioning that radix entomolaris seems to be more frequently detected in individuals of Chinese ancestry, encompassing those with Mongolian, Taiwanese, and Korean heritage. Radix entomolaris (RE) is higher in Eastern Asian societies, while its occurrence is relatively lower in other populations, such as Europeans [4,5]. According to existing literature, radix entomolaris in African groups has been documented at a rate of 3% [6].

In contrast, in Eurasian and Indian cultures, its prevalence is less than 5% [7]. Research undertaken on populations displaying Mongolian features, including Chinese, Eskimos, and American Indians, has documented the occurrence of radix entomolaris at varying prevalence rates, ranging from 5% to 30% [8]. The greater prevalence of radix entomolaris among the populations mentioned above is considered a naturally occurring anatomical trait. Radix entomolaris in individuals of Caucasian descent is not commonly observed, so the reported incidence rates of 3.4% and 4.2% within this population are deemed unusual [3,9].

The research investigation ascertained that the prevalence of radix entomolaris in the mandibular first molar of female patients was 6.92%, a substantially greater proportion as compared to the observed occurrence in male patients (5.83%) [8,10]. The mesiobuccal side of the tooth has been discovered to exhibit the presence of an extra root, referred to as the radix paramolaris (RP). Radix paramolaris (RP) is relatively infrequent and small in size, exhibiting a lower incidence rate when compared to radix entomolaris [11]. According to a study by Visser et al. [12], radix paramolaris was found in the first lower molar, second molar, and third molar at rates of 0%, 0.5%, and 2%, respectively. In another study, a 12-year-old female patient was diagnosed with radix entomolaris (RE) and radix paramolaris (RP) in the lower first molar [13]. 

Two-dimensional periapical radiography is a frequently used diagnostic imaging technique for the evaluation of root morphology and the detection of probable apical lesions. However, several restrictions on routine periapical radiography could prevent a correct diagnosis of periapical pathological lesions or the delivery of the necessary care [14,15].

Cone beam computed tomography (CBCT) is a medical imaging technique that comprehensively visualizes anatomical structures and pathological conditions. These anatomical structures can be achieved by generating multi-planar reconstructed images, which provide views in sagittal, axial, coronal, and diagonal orientations [16]. Examining dental canal morphology across diverse groups yields valuable clinical insights that can enhance patient treatment outcomes [17]. However, many dental professionals lack knowledge regarding dental anatomical structures, as they have challenges in accurately diagnosing them due to the limitations posed by photographs captured at improper angles. Therefore, the objective of this research is to assess the frequency and morphological characteristics of radix entomolaris (RE) and radix paramolaris (RP) in mandibular molars by utilizing cone-beam computed tomography (CBCT) imaging. The study focuses on a specific population residing in the northern region of Iran during the years 2020 and 2021. 

## Materials and methods

The present study employed a cross-sectional descriptive-analytical design. A systematic random sampling method was employed to select CBCT stereotypes of lower first and second molars from an oral-maxillofacial radiology center from 2020-2021. 

Ethical approval

Ethical approval was obtained from the hospital's research department with code number IRB.MAZUMS.REC.1400.256.376. 

Procedure

The scans on the permanent lower first and second molars revealed that the teeth exhibited complete development with closed and mature apical ends. Images depicting decaying or calcified canals in the first and second molars, as well as those displaying past root canal treatment and post-cementation, were included. Images without sufficient precision were eliminated from the analysis. The cone beam computed tomography (CBCT) pictures were acquired using the Newton GiANO system (QR Verona, Verona, Italy) by specialists in oral and maxillofacial radiology. Subsequently, the images were analyzed using the NNT viewer software.

The photos were analyzed by two specialists, namely a radiologist and an endodontist. The images were subsequently forwarded to another endodontist for review to address potential ambiguities. The images were analyzed using the highest available resolution and contrast. Additional roots, if they were present, were documented within two distinct groups known as entomolaris and paramolaris. The software was utilized to capture the radix type, patient's gender, radix root canal length, and radix root curvature for photos containing radix. 

The investigation of radix root canal anatomy was conducted utilizing Vertucci's categorization type I, as a previous study claimed that Vertucci’s categorization failed to fit in each sample [18]. The measurement of radix root curvature used the following procedure: First, a line was delineated along the tooth's longitudinal axis, originating from the orifice. Subsequently, the point at which the canal deviated from this line was identified and labeled as point A. Last, a second line was drawn from the apical foramen to point A. The measurement of the angle between these two lines serves as an indicator of the curvature angle. The Seidenberg [19] classification categorizes root curvature into three distinct groups: mild curvature, which is defined as being less than 5 degrees; moderate curvature, which falls within the range of 5 to 25 degrees; and severe curvature, which spans from 25 to 70 degrees.

The data analysis employed descriptive statistical approaches, utilizing a frequency table to summarize the results. The chi-squared test was utilized to examine the association between the underlying and independent variables regarding the occurrence of radix. A multiple logistic regression analysis was conducted at a significance level of 5% to simultaneously account for factors and assess the impact of each variable on the chance of a radix occurrence. The statistical analysis was conducted using IBM Corp. Released 2017. IBM SPSS Statistics for Windows, Version 25.0. Armonk, NY: IBM Corp. 

## Results

This cross-sectional study examined 376 CBCT images of teeth in an Oral and Maxillofacial Radiology Center Archive in Sari, Iran. In this case, 1504 left and right mandibular molars were examined. The results indicated a 0.74% prevalence of radix among people who went to an Oral and Maxillofacial Radiology Center in Sari. All radix roots were seen in the lower first molar; in addition, the prevalence of radix in the mandibular second molar was 0%. Table 1 reports the prevalence of entomolaris and paramolaris in patients who had radix roots. The prevalence of radix was found to be 0.74%, with entomolaris and paramolaris present in 54.54% and 45.46% of cases, respectively. There was no statistically significant difference between the two variables of radix type and gender, as indicated by a p-value of 0.08. The mean curvature and length of the radix root were measured to be 56.63° and 15.09 mm, respectively.

**Table 1:** Entomolaris and paramolaris frequencies in patients

Studied variable	Entomolaris		Paramolaris	
	Frequency	Frequency (%)	Frequency	Frequency (%)
Molar teeth	6	54.54	5	45.46

Table 2 reports the frequency of entomolaris and paramolaris among men and women who have molar radix roots.

**Table 2:** Frequency of radix in patients based on their gender

Studied variable		Men		Women		Sig.
		Frequency	Frequency (%)	Frequency	Frequency (%)	
Molar teeth	Entomolaris	5	83.3	1	20	0.08
	Paramolaris	1	16.7	4	80	
	Total	6	100	5	100	

The average amount and standard deviation of root curvature in patients with radix equaled 56.63 and 10.13, respectively, while the mean value and standard deviation of root length of patients with radix equaled 15.09 and 1.57, respectively. The study found that the root curvature of entomolaris exhibited a statistically significant difference compared to paramolaris (P=0.031). The mean length of the radix entomolaris and paramolaris roots was found to be 15.50 and 14.60 mm, respectively.

Table 3 reports the mean value and standard deviation (SD) for root curvature in patients who had radix entomolaris and radix paramolaris. Results indicate a significant statistical difference between the mean values of root curvature regarding the type of radix. 

**Table 3:** Mean value of root curvature in patients based on the radix type

Radix profile		Root curvature		Sig.
Variable	Frequency	Mean	SD	
Entomolaris	6	62.33	7.86	0.031
Paramolaris	5	49.80	8.49	

Table 4 reports the mean value and standard deviation of root length in patients with RE and RP. Moreover, average root length indicated no significant difference between the two types of radixes. The mean length of the radix entomolaris and paramolaris roots was found to be 15.50 and 14.60 mm, respectively. There was no statistically significant difference observed in the mean root lengths of the various radix types (P = 0.37). It was reported that type 1 Vertucci's classification was observed for 100% of the root canal anatomy of radixes. 

**Table 4:** Mean value of root length in patients based on the radix type

Radix profile		Root curvature		Sig.
Variable	Frequency	Mean	SD	
Entomolaris	6	15.50	1.87	0.37
Paramolaris	5	14.60	1.14	

## Discussion

The failure of root canal therapy can be attributed to various factors, including missed canals and inadequate cleaning and shaping techniques. It is crucial to consider the morphology and quantity of roots, as these factors significantly impact the procedure's outcome and ensure successful root treatment. Typically, the lower first and second permanent molars exhibit a mesial root and a distal root. Under very extraordinary circumstances, however, it is possible for a third supplementary root known as the radix to manifest due to variations in ethnic background or dental architecture. The radix root is predominantly found in the distolingual entomolaris and paramolaris, with rare instances of development in the mesiobuccal [1,2]. Cone-beam computed tomography (CBCT) is a diagnostic imaging technique that comprehensively visualizes anatomical and pathological features. These structures are achieved by reconstructing images in many planes, including sagittal, axial, coronal, and diagonal views [20]. This study presents findings on the prevalence and anatomical characteristics of radix entomolaris (RE) and radix paramolaris (RP) in a sample of 1504 mandibular molars from a population in North Iran, utilizing cone-beam computed tomography (CBCT) images.

The current study found a radix incidence of 0.75% among patients. These results are much less than the study conducted by Sperber et al., revealing a radix prevalence of 3.12% in 480 removed mandibular molar teeth of the African population [6]. Generally, it appears that there are variations in the prevalence ranges seen by different races, countries, and geographical places. For example, Ferraz, Pecora, and Fabian [6,20] documented a prevalence rate of 1.6% and 4.2% for the third root or radix among Germans and Brazilians, respectively. Trautman conducted a study on Asian communities and presented the prevalence of radix as follows: Malays (5.3%), Chinese (7.9%), Singaporeans (10.5%), and Indonesians (17.2%) [7]. Ferraz & Pecora [20] have documented a prevalence range of 1% to 8.2% for radix among the Mongols of the Malay population. Ferraz and Pecora [20] conducted a study on the prevalence of radix in the black population. Their findings indicated that Madagascans and Brazilians exhibited radix prevalence rates of 5% and 2.6%, respectively. In a separate investigation conducted on a population exhibiting Mongolian characteristics, such as individuals of Chinese, Eskimo, and American Indian descent, findings revealed a prevalence rate of 5%-30% for radix entomolaris [8]. 

In the present study, entomolaris was observed at a frequency of 54.54%, while paramolaris was found at a frequency of 45.46%. Consequently, the prevalence of entomolaris was more substantial than the prevalence rate of paramolaris. Our results are comparable with the study of Zhu et al. [21], in which they revealed a prevalence rate of 21.6% for the third root among the Chinese population. Their study also revealed that the third root is predominantly distal or entomolaris in most cases, specifically 68.3%, which aligns with the findings of our investigation [21]. In contrast, a study conducted by Hassan AL-Alawi et al. (2019) examined a population in Saudi Arabia and documented prevalence rates of 4.5%, 4.3%, and 0.3% for radix molaris (RM), radix entomolaris (RE), and radix paramolaris (RP), respectively [22]. A study conducted by Maryam Kuzekanani examined the prevalence of radix entomolaris throughout Iranian society and found a rate of 6%, which was higher than the prevalence rates observed in European and Caucasian groups (less than 2%) [11,23]. Hosseini et al. [24] analyzed 200 CBCT images obtained from individuals in Babol, Iran. The researchers observed a prevalence rate of 3% for radix entomolaris (RE) within this specific group. All cases of radix entomolaris exhibited a buccolingual crown, and our study results indicated no significant association between radix entomolaris and gender, therefore aligning with our findings [24]. 

Current study reported the presence of three mid-mesial canals in women, whereas men were found to have only one mid-mesial canal. There was only one radix that existed for both males and females. Two mesial canals were identified bilaterally, with both radix roots on the right side and the absence of the radix root on the left side. In a previous study, the presence of a mid-mesial canal and radix root did not demonstrate any correlation between tooth location and sexual companionship [16]. The present investigation also corroborated this finding, as it observed no statistically significant distinction between the two variables of radix type and gender. Hence, the presence of paramolaris and entomolaris is not contingent upon gender.

The current investigation revealed that all radixes exhibited type 1 root canal anatomy, as classified by Vertucci, with a prevalence of 100%. Following the findings of our survey, Bains et al. conducted a case study that reported one root canal with Type I classification according to Vertucci's classification system [25]. Kuzekanani et al. [11] reported their findings on classifying entomolarises in the mandibular first molar. The study identified 10 cases classified as Type I and five as Type II. Mukhaimer et al. [26] conducted a comprehensive examination of a sample of 320 roots located within the geographic region of Palestine. The distribution of Vertucci types in mesial roots was seen to be as follows: Type I (1.15%), Type II (38.8%), Type III (1.9%), Type IV (53.8%), and Type V (1.15%). Other categories demonstrated lower frequencies. The previous study observed that the distribution of Vertucci types in distal roots exhibited the highest prevalence for Type I (57.5%), followed by Type II (22.5%), Type III (10.6%), Type IV (8.1%), and Type V (1.3%). Additional types were shown to possess rather low frequencies [26]. Martins et al. examined 11892 teeth and found radix prevalence equaled 2.2% and 2.7% in the first and second molars, respectively. They observed 14 entomolaris cases, of which four occurred in the second molar. In the examined mandibular first molars, 93.4% of two-rooted teeth in the mesial root were Type I of Vertucci's classification, and 70.9% in the distal root were Type I [27]. The aforementioned studies did not express the type of Vertucci's classification only for radix roots. However, our study reported that type 1 of Vertucci’s classification occurred in 100% of studied roots, and it is justifiable because the paramolaris root is mesial, and the entomolaris root is distal. Furthermore, the average angle of root curvature in entomolaris was observed as 62.33± 7.86, while in paramolaris it was observed as 49.80 ± 8.49. The current study reported severe curvatures when compared with the previous study by Qiao et al. [21], in which they found a RE root curvature of 40.69± 14.37° in 487 mandibular first molar cases.

Limitation of the study

One of the limitations inherent in this study is the inability to assess the radix root in both right and left molars symmetrically for each participant, as it is ethically unacceptable to conduct imaging procedures on patients without a valid clinical indication. Another potential drawback of the study is its exclusive reliance on photos obtained from a single radiology center's archive. It would have been advantageous to include images from many radiology centers within the same province to enhance the generalizability of current findings. Another constraint is the small sample size, potentially adversely influencing the outcomes.

## Conclusions

The prevalence of radix root is not widespread in the northern regions of Iran. Typically, the prevalence of entomolaris exceeds that of paramolaris. Entomolaris is higher in males, whereas paramolaris is more commonly observed in females with radix roots. There was no statistically significant difference seen concerning radix type or gender. The mean curvature of the radix entomolaris root was much higher than that of the radix paramolaris. The mean length of the RE root was seen to be greater than that of the RP root; however, this disparity did not reach statistical significance. All radices were classified as Type I according to Vertucci's classification. It is recommended to undertake a systematic literature review to examine the precise correlation between the prevalence of radixes and various ethnicities, as well as identify all elements that are associated with radixes and determine the average range of radix profiles.

The research undertaken within the geographical area exhibits variations, thus suggesting the need for scientists to conduct additional studies, including larger sample sizes, to accurately determine the incidence of radix in different ethnic groups. Several studies have explored the correlation between ethnicities and radixes, although no research has investigated the underlying reasons for this association. Hence, it is recommended to undertake this study to attain scientific validation for its pursuit. 
